# Advancing Climate Mitigation, Adaptation, and Equity Simultaneously: The Transformative Potential of Investments in Gender Equality

**DOI:** 10.3389/phrs.2026.1607423

**Published:** 2026-02-10

**Authors:** Jody Heymann, Aleta Sprague, Abena D. Oduro, Laurel Grzesik-Mourad

**Affiliations:** 1 WORLD Policy Analysis Center, Fielding School of Public Health, University of California, Los Angeles, Los Angeles, CA, United States; 2 University of Ghana, Accra, Ghana

**Keywords:** equality, gender, climate change mitigation, climate change adaptation, policy

## Abstract

**Background:**

Climate change negotiations often stall because of debates about equity. The SDGs affirm all countries’ responsibilities to act on climate and high-income countries’ initial $100 billion annual financing commitment; the SDGs also affirm fundamental human rights that are foundational to both equality and a strong economy. Nevertheless, climate investments historically have neglected people-centered climate solutions that would powerfully advance these interconnected goals.

**Analysis:**

Realizing girls’ equal rights in education, women’s equal rights at work, and freedom from gender-based violence would fulfill fundamental human rights while markedly accelerating climate mitigation and adaptation. Mechanisms include increased reproductive autonomy, higher adoption of sustainable fuels and regenerative agriculture, increased resilience to climate disasters, and greater gender parity in leadership.

**Policy Options:**

A variety of options are available for countries to invest in gender equality simultaneously with nature- and energy-based solutions. These include carbon markets, debt-for-equity swaps, and existing UNFCCC financing mechanisms. The climate impacts of people-centered solutions are estimable.

**Conclusion:**

Successfully addressing climate will require investments in gender equality. Bilateral and multilateral bodies can build on existing data to achieve this through a variety of climate mechanisms.

## Background

Advancing equal rights has been central to global goals since the founding of the United Nations. The very first line of the Universal Declaration of Human Rights, adopted in 1948, proclaims unequivocally that “the equal and inalienable rights of all members of the human family is the foundation of freedom, justice and peace in the world.” Likewise, advancing equality is fundamental to nearly all of the Sustainable Development Goals (SDGs) agreed to by 193 countries in 2015, while two goals are dedicated solely to equality: SDG 5, “Achieve gender equality and empower all women and girls,” and SDG 10, “Reduce inequality within and among countries.”

Realizing equal rights is essential because of their intrinsic value—but it’s also a prerequisite to overcoming major global challenges. Among the most urgent is climate change: inequalities within and across countries have stymied progress on meeting IPCC targets. Climate change talks and agreements have often stalled amidst debates about equity, with low-income countries emphasizing their need for economic growth and the responsibility of high-income countries—which have historically been the largest emitters and have grown as a result—to cover the costs of mitigation and adaptation [1].

The urgent need is for all countries to mitigate and adapt going forward and for lower-income countries to still be able to grow, while those with the most emission-driven growth support the costs. The SDGs provide a framework for this by simultaneously: 1) holding all countries accountable for combatting climate change while holding wealthier countries accountable for markedly increasing available funding (SDG 13), and 2) urging and supporting all countries to realize fundamental human rights that are foundational to both equality and a strong economy, including access to nutrition (SDG 2), health (SDG 3), education (SDG 4), and decent work for all people (SDG 8).

Nevertheless, climate investments historically have not supported these interconnected goals, instead prioritizing investments in technology. Indeed, technology is crucial to mitigation and to a lesser extent may help with adaptation. But neither can be solved without investments in people. Carbon emissions are the product of emissions per person and the number of people. The human elements that shape the impact of mitigation efforts include both -popularly recognized factors such as what energy people are able to access and choose to use, as well as less widely recognized factors such as how people farm, whether people live free from violence, and whether they have reproductive autonomy. Similarly, whether people have the time, resources, and information to adapt to the risks and conditions of climate change will shape both households’ and countries’ resilience. Moreover, while investments in people-centered climate solutions have been insufficient overall, investments in women and girls—who are the disproportionately affected by climate change across countries—have been even lower.

This policy brief argues for more action and investment to support gender equality, a long-established fundamental right that is foundational to successful human solutions to climate change mitigation and adaptation. The brief will: 1) provide an overview of some of the evidence that advancing gender equality can accelerate mitigation, 2) illustrate how gender equality matters to adaptation, and 3) describe what taking gender equality seriously would mean for the global and regional mechanisms currently designed to address climate change.

The evidence and recommendations in this policy brief are based on an in-depth review of the evidence relevant to approaches that can address urgent climate mitigation and adaptation needs and simultaneously advance equal opportunity. We first mapped potential pathways for climate change mitigation and adaptation. We then conducted a comprehensive literature review to evaluate potential approaches that would both accelerate climate mitigation and adaptation and advance equity goals. Given that previous scientific consensus efforts have highlighted the importance of gender equality in education and health to climate mitigation, we paid particular attention to the research evidence on the full range of mechanisms with respect to gender equality. Finally, we ran test simulations to determine the feasibility of estimating the climate impact of gender equality interventions with similar rigor and methods to those used for other interventions included in climate markets and funding.

## Analysis

### Reducing Emissions

Investing in education has long been recognized as a powerful engine for equity and development. The World Bank estimates that if girls’ educational attainment were equal to that of boys, global human capital wealth could increase by US$ 15 trillion to US$ 30 trillion, while UNICEF has shown that child mortality would be cut in half if girls completed secondary education—and these are just two examples of many. Yet education also has a powerful—and less recognized—role to play in mitigation and adaptation. A large global network of scientists and researchers focused on climate change solutions estimated that universal access to education and reproductive health would reduce greenhouse gas (GHG) emissions by nearly 70 gigatons by 2050, with 55% of the impact from low- and middle-income countries and 45% from high-income countries [2]. To put these impacts in context, the initiative forecasted that universal education and voluntary family planning would lead to a greater reduction in carbon emissions by 2050 than:

- All transportation solutions combined (including electric vehicles, decarbonizing aviation, and decarbonizing shipping).
- Deploying both onshore and offshore wind turbines.
- Tropical forest restoration [3].

To be clear, energy and nature-based solutions are critical—and governments and industries must reduce dependence on fossil fuels. At the same time, these investments need to be complemented by human investments. Research suggests that investments in reducing gender inequality in education and work and preventing GBV would all advance mitigation. In this section we survey the mechanisms and evidence, with particular attention to girls’ education as one powerful example.

#### Impacts on Reproductive Autonomy

Access to education increases girls’ health knowledge, access to services, and autonomy in decision-making [4–6]. Young women with greater educational attainment, on average, also express a preference for delaying childbearing and having smaller families [7]; some evidence likewise suggests lower fertility preferences for men who stay in school longer [8], or whose wives have more education [9]. Access to education also reduces child marriage [10], which is a major threat to girls’ health and opportunities and a key driver of adolescent pregnancies. Recent estimates from UNESCO suggest that achieving universal secondary education would virtually end child marriage—and that ending child marriage would lower fertility rates by a third, as girls are able to stay in school and reach adulthood before starting a family [11].

Gender equality in other spheres likewise matters to reproductive autonomy—a fundamental right on its own. A study of 23 African countries found that laws prohibiting domestic violence reduced women’s unmet need for family planning by over 20% [12]. At the same time, young women’s greater access to employment opportunities has been associated with a desire to delay childbearing and have fewer children, coupled with a desire to pursue more education and training and continue working after marriage [13, 14].

Importantly, reproductive autonomy includes the right to make independent decisions about family size, whether that means fewer or more children [15]. In practice, however, the greater threat to women’s reproductive autonomy globally is the inability to decide to have fewer children or to have children later in life [16]. Realizing this fundamental right in turn benefits climate, as delayed births and fewer unwanted pregnancies lower emissions by slowing population growth [17, 18].

#### Impacts on Fuel Use

Higher educational attainment also leads to higher incomes. The impacts on emissions depend on how higher income is used. One case in point is fuel use.

Biomass and waste burning—which create substantial carbon emissions while also threatening human health through their impacts on air quality—account for a majority of household energy consumption in Africa, as well as substantial shares in small island nations, South and Central America, and Asia [4]. However, evidence suggests that greater educational attainment can contribute to households adopting more sustainable fuels. For example, a systematic review examining factors that shape adoption of improved cookstoves and/or cleaner fuels for cooking found that higher educational attainment and higher income were associated with higher uptake in nearly all studies [19]. Moreover, women are generally more likely than men to choose clean fuels [20], and take-up among women with greater education is even higher [21]. Women’s equal opportunities in employment can likewise support greater uptake of these technologies by increasing women’s income [22].

#### Impacts on Agricultural Practices

More sustainable agricultural practices have a powerful role to play in reducing emissions, preserving biodiversity, and ensuring food security. Studies from a range of countries have found that higher educational attainment is associated with higher take-up of regenerative agricultural practices.

In Brazil, for example, farmers with higher levels of education were more likely to adopt crop-livestock integration [23]. In Pakistan and Ethiopia, more educated women farmers were more likely to adopt a range of conservation and climate-smart agricultural (CSA) practices than women with less education [24, 25]. Across Malawi, Mozambique, and Zambia, research found that one additional year of formal education increased the likelihood of a farmer adopting land, soil, and water conservation measures by 13% [26]. Notably, while studies find increased uptake of sustainable agricultural practices among farmers with higher levels of education regardless of gender, in some countries, men’s uptake is higher, potentially due to men’s greater access to resources—underscoring why reducing barriers to education for all must be an imperative for both equity and climate.

#### Impacts on Leadership and Climate Action

Girls who are able to complete their education have greater opportunities to take on public and private sector leadership roles as adults. Likewise, women who face less discrimination at work and lower risks of violence at home are more likely to be able to attain and retain leadership positions. Greater gender equity in leadership in turn supports more climate-conscious decision-making.

For example, studies spanning a wide range of countries have documented a positive relationship between more gender-equal representation in parliament and lower carbon emissions [27]. A review of studies focused on the private sector likewise found that greater gender equity in top positions was associated with firms achieving higher environmental performance ratings and providing more transparent sustainability disclosures [28]. Currently, however, just 31% of managers, 7% of Fortune Global 500 CEOs, and 25% of national parliamentarians globally are women—illustrating the significant untapped potential for impact on both gender equity and climate action [29].

### Supporting Resilience to Climate-Related Disasters

Alongside mitigation, all countries must adapt. Across countries, climate-related disasters are already causing billions of dollars of damage each year, and disproportionately harming economically vulnerable communities. Yearly rankings of countries’ vulnerability to climate change consistently report that LMICs in Africa, alongside small island nations, are facing the greatest collective risks across six key dimensions: food, water, health, ecosystem service, human habitat, and infrastructure [30]. The scale and intensity of wildfires, floods, and other climate disasters are also increasing in high-income countries, where they disproportionately affect women and marginalized communities [31]. Higher educational attainment has been linked with higher rates of survival following natural disasters. Greater access to education enhances access to information and is associated with higher levels of disaster preparedness, as well as better access to support and resources following a disaster [32, 33]. Educating girls may be particularly impactful: one analysis estimated that increasing the share of girls finishing lower secondary school in sub-Saharan African from roughly 30%–70% would reduce deaths from climate disasters between 2040 and 2050 by 60% [34]. Greater household income--facilitated by women as well as men having access to employment---also improves resilience following disasters [35].

## Policy Options

### Increasing Climate Investments in Gender Equality

Some crucial policy changes that matter to climate and equality can be made without substantial funding; modest resources are needed to ensure successful implementation. Examples include ensuring that it is illegal to discriminate based on gender in hiring, promotion, and other aspects of work. Currently 84 countries lack at least one of these basic protections [36].

Advancing equality in other areas will require investment. The cost of tuition for secondary education is a major obstacle to the attendance of girls and low-income children [10, 37]. Yet 57 countries do not provide tuition-free secondary education [36]. The benefits of girls’ education to national economies will make it affordable for governments to fund secondary education themselves in the long run. In the short run, weak economies and limited budgets are obstacles for a number of countries—and supporting girls’ education with funds from the mechanisms below could help countries turn a corner on emissions and on equality.

Given its powerful impact on climate mitigation, girls’ education should receive climate funds as well as other sources of funding. Government-operated compliance markets are currently valued in the hundreds of billions, and continue to grow [38, 39]. If gender equality in education received the same portion of the voluntary and regulatory carbon market as the percentage of emissions it could reduce, this could lead to a 50-100X increase in financing.

Investing in girls’ education should be part of the $100 billion initial global commitment in SDG 13 to increase funds available to low-income countries to mitigate and adapt, which was since updated to $300 billion at COP29. Moreover, given that many of the countries with high numbers of girls out of school are also highly indebted, debt-for-equity swaps modeled on current debt-for-nature swaps hold potential to provide significant additional return on investments. Community-based programs could be part of the voluntary market.

### Data on Climate Value of National Policies and Programs

Investments in girls’ education, ending child marriage and GBV, supporting gender equality at work and in leadership should not be limited to their “carbon value.” Each is invaluable in its own right because of how it can directly transform the quality of life of half of the people on the planet—and in turn the health and wellbeing of their children, partners, parents, and communities.

At the same time, carbon investments should not ignore the value of gender equality as a carbon solution and carbon investments can be an important funding source because of how high the carbon value is. The 70-gigaton reduction in emissions that scientists estimated gender equality would lead to is itself likely an underestimate. While it considered the impacts of girls’ and women’s greater reproductive autonomy on countries’ resource use [2], this estimate did not factor in the range of other mechanisms by which girls’ education can powerfully support climate change mitigation and adaptation. Among these are higher adoption of sustainable fuels, increased resilience to climate disasters, higher take-up of regenerative agriculture, and greater gender parity in leadership, which may increase climate action by companies and governments.

The global estimate of climate benefit will need to be broken down to country- and program-level estimates. While there will be some challenges, estimating the carbon value of advancing gender equality is no more difficult than estimating the value of land preservation. Take increasing girls’ graduation from secondary school as an example. Just as there have been multiple estimates of the global impact of gender equality in education on GDP growth [40, 41], we can estimate the impact of increasing girls’ graduation on carbon emissions. These estimates can be made robustly by examining how carbon-relevant outcomes have changed in countries that lower barriers to girls’ education versus countries that do not. The same can be done with reducing workplace discrimination, child marriage, and domestic violence because we have longitudinal data on these policies [36]. Combining this with outcome data on many of the drivers of carbon emissions at a household level, we can estimate the distribution of both the environmental benefits and the implementation costs of each policy at the country level.

This would allow low-income countries to apply to carbon funds to increase the resources available to them to make secondary school tuition-free, or to invest additional resources in programs to end child marriage and GBV and support equal opportunity at work.

## Conclusion

Both current climate realities and long-term trajectories make clear that all countries need to mitigate and all countries need to adapt. Doing so equitably necessitates increasing investments in solutions that not only reduce emissions and facilitate resilience but also level up access to opportunities, economic resources, and health across countries and across populations. Gender equality is central amongst these.

At present, however, the climate funding going to gender equality has largely been symbolic, despite the fact that investments in gender equality can yield both short- and long-term gains in climate mitigation, as well as for adaptation. It’s long past time for substantial investments to be made in supporting gender equality, which can powerfully advance climate mitigation and adaptation and simultaneously improve economies, human health, and intergenerational wellbeing. Ensuring girls have an equal chance to complete secondary education and beyond, that women have equal opportunities at work, and that all women and girls can live free from violence is long overdue, and the reasons to take action are as numerous as they are pressing.
