# Correction: Advancing Climate Mitigation, Adaptation, and Equity Simultaneously: The Transformative Potential of Investments in Gender Equality

**DOI:** 10.3389/phrs.2026.1609713

**Published:** 2026-03-20

**Authors:** Jody Heymann, Aleta Sprague, Abena D. Oduro, Laurel Grzesik-Mourad

**Affiliations:** 1 WORLD Policy Analysis Center, Fielding School of Public Health, University of California, Los Angeles, Los Angeles, CA, United States; 2 University of Ghana, Accra, Ghana

**Keywords:** equality, gender, climate change mitigation, climate change adaptation, policy

The affiliation “University of Ghana, Accra, Ghana” was omitted for author Abena D. Oduro. This affiliation has now been added for author Abena D. Oduro.

Author Abena D. Oduro was erroneously assigned to affiliation WORLD Policy Analysis Center. This affiliation has now been removed for author Abena D. Oduro.

The original article has been updated.

